# Effectiveness of a social inclusion program in people with non-affective psychosis

**DOI:** 10.1186/s12888-018-1728-5

**Published:** 2018-06-07

**Authors:** Fausto Mazzi, Flavia Baccari, Francesco Mungai, Manuela Ciambellini, Lisa Brescancin, Fabrizio Starace

**Affiliations:** 1 Department of Mental Health and Drug Abuse, AUSL Modena, Viale L Muratori 201, 41124 Modena, Italy; 2 Social Point Modena – Centro Servizi Volontariato, Modena, Italy

**Keywords:** Social inclusion, Psychosis, Recovery, Self-stigma, Disability, Quality of life, Psychosocial interventions

## Abstract

**Background:**

People with psychotic illness suffer from reduced quality of life and often from an insufficient level of social inclusion. These variables are associated with several negative outcomes, such as higher neuro-cognitive deficits, negative symptoms, internalised stigma, increased cardiovascular risk and, most importantly, excess mortality. To date, only a minority of social interventions in psychosis have been investigated. Since 2011, the Department of Mental Health and Substance Abuse in Modena introduced the “Social Point” program, which provides social inclusion interventions to promote active social participation for patients suffering from severe mental illness.

The aim of this study was to assess whether a social inclusion intervention is associated with better outcomes in terms of personal and social recovery, with particular reference to the areas of social functioning and activity, and subjective dimensions such as self-esteem, self-stigma and perceived quality of life.

**Methods:**

A cross-sectional design was adopted to compare 30 subjects, selected at the completion of “Social Point” program, with a group of subjects, matched for socio-demographic and clinical features, selected from a wait list for “Social Point”. All subjects were evaluated by means of instruments assessing: level of disability, level of functioning, severity of psychopathology, self-esteem, internalised stigma and quality of life.

**Results:**

Overall, the results of the study suggest that social inclusion interventions may be effective in people suffering from non-affective psychosis. A dose-effect relationship was also found between higher number of activities per patient and better outcomes within both social and psychopathological domains. However, due to the cross-sectional design of the study no definitive causality can be inferred.

**Conclusion:**

Psychosocial interventions promoting social inclusion are likely to represent an effective approach to improve personal and social recovery.

## Background

Despite the lack of unanimous definition in the literature, social inclusion can be generally conceptualised as the opportunity for the individual to participating in key functions or activities and in economic, social and cultural life of his/her community, exercising the rights of his/her citizenship and enjoying an adequate standard of living and wellbeing [1].

People suffering from psychosis experience lower quality of life and reduced social inclusion [2, 3]. They are more likely to live alone [4], be unemployed [5], have fewer significant relationships [6] and are at high risk of experiencing “social disadvantage” [7]. Moreover, available evidence indicates that social isolation represents a risk factor for cardiovascular diseases [8] and a major cause of excess mortality [9]. Social disadvantage and psychosis are tightly intertwined making it difficult to disentangle their cause-effect relationship. On one hand, having fewer social relationships may be the result of psychosis and of negative symptoms in particular, such as emotional and social withdrawal, low level of energy and motivation, which considerably interfere with the ability to form and maintain a social network [10]. On the other hand, social disadvantage and discrimination suffered by psychotic patients, such as unemployment, loss of productivity and societal role, may reduce the skills and opportunities necessary to form and maintain supportive social networks. This vicious circle can, in turn, generate low self-confidence/self-esteem and internalised stigma, which interfere with satisfactory social functioning [5, 11]. Furthermore, smaller social networks associate with worse outcomes such as increased risk for hospital admissions [12], poorer functioning and worsening of symptoms in subjects suffering from schizophrenia [4].

Indeed, social inclusion interventions represent a milestone in the process of recovery as they aim at facilitating psychological adaptation [13]. Improving social networks correlates with better physical and mental health outcomes, both in affective disorders [14, 15] and psychoses [16]. Stable relationships, living with someone and being employed may also have a protective role [7]. Interventions aimed at increasing social participation in people with mental health problems can effectively increase sense of belonging and reduce psychological distress [17, 18]. In addition, social interventions can improve the overall engagement with mental health services [19]. Therefore, improving social outcomes in psychosis represents a paramount objective for mental health services in terms of economic sustainability and ethical implications [20].

An additional aspect worth considering more in depth is the impact of public and internalised stigma on people with mental health problems [21]. Over-concern with failure and rejection, experienced by psychotic patients can give raise to dysfunctional attitudes [22] characterized by avoidance and lack of motivation. These attitudes, in turn, can lead to social and vocational disengagement [23]. The internalization of negative societal stereotypes is associated with self-stigma, whose consequences often translate in the so called “why try” effect [24, 25]. Social interventions in mental health should necessarily consider the internalised stigma as a major target. Self-stigma is expressed by over-concern with societal prejudices towards mental illness and mental health service users. Internalised stigma has been reported to be closely related to long term outcomes of personal and social recovery [26]. The hypothesis formulated is of a close relationship between internalised stigma, level of activity and social inclusion [27, 28]. The experience of stigmatization, despite interfering with recovery, tends to be rather subjective in nature and does not always reflect an actual experience of discrimination [29–31]. Furthermore, it is possible that even within stigmatizing contexts, people may feel indifferent or even being prompted to react positively boosting their self-confidence and self-esteem [32, 33]. Reaction to stigma in mental health represents a major predictive factor for personal and social recovery. Interventions aimed at modifying negative subjective beliefs on mental illness may prompt individuals to activate or develop resources and social skills necessary to achieve a full recovery.

Finally, social interventions in mental health should encompass patients’ subjective views and preferences, consider their needs and diversity and engage with community and voluntary sectors to provide a broad range of services as opposed to a “one-size-fits-all” approach [34].

To date, only a minority of social interventions in psychosis have been investigated to evaluate their impact on level of activity, quality of life, to assess their effectiveness in improving internalised stigma, reducing negative attitude and perception of patients towards their mental illness [35, 36]. Developing an evaluation system able to assess different interventions represents a major challenge, as a wide range of approaches can be used to promote social inclusion. Nevertheless, systematic research on social intervention indicate that studies using experimental designs, representative samples and sufficient follow-up length are deemed necessary to support the evidence and using it to make recommendation to mental health policies [17].

Since 2011, the Department of Mental Health and Substance Abuse in Modena has implemented the program “Social Point”, which provides social inclusion interventions and promote active social participation for patients suffering from severe mental illness.

The program has three main objectives:

1. social inclusion facilitation of people with mental health problems via volunteering and leisure activities, promoting and enhancing individual and collective resources.
2. awareness raising campaign, training and information within the community on mental health issues.
3. spreading positive ideas on mental health as a matter of collective responsibility with the potential to create social capital for the community.

In line with the principles of participation and citizenship, and pursuing inclusion and empowerment of service users, Social Point program collaborates with the local authorities to build “collective projects”, actively involving both people with mental health problems and the general population. Besides, personalized “care pathways” are guaranteed and built on personal views and preferences of service users. Social Point workers are called to facilitate processes leading to change and ultimately to recovery, rather than pursuing standardized projects. The areas of intervention covered by “Social Point” encompass: advice and awareness on mental illness, education and skills development, one-to-one and group sessions of self-help, physical and leisure activities.

The experience gathered so far has shown that even users with the most severe psychopathological conditions can greatly benefit from individualized rehabilitation projects, which can change the perception of the social context towards them, via the development and recognition of abilities, resources and proactive attitudes.

The aim of this study was to assess, in a comparative cross-sectional design, whether the social interventions provided by “Social Point” associate with better outcomes in the areas of personal and social recovery, with particular focus placed on social functioning and level of activity, and subjective measures in the areas of self-esteem, self-stigma and perceived quality of life of subjects under the care of the mental health services in Modena.

## Methods

### Sample

Thirty subjects, under the care of the mental health services in Modena, were recruited for the study as a representative sample of patients with non-affective psychosis who participated to social inclusion activities delivered by “Social Point” between 1st January 2011 and 1st January 2014. The outcome measures of the sample, collected and analysed at the end of the intervention, were compared with a group of subjects selected from a wait list for “Social Point”, matched for socio-demographic and clinical features: gender, age, civil status, education level, employment, economic status, diagnosis, pharmacological treatment, duration of illness, number of previous hospital admission, pre-morbid social functioning, social disadvantage.

Data were collected by means of the Case Register of the Mental Health Department of Modena, integrated with additional information gathered from the patients’ clinical notes and the responsible clinician at the local community mental health service.

Out of the total eligible sample, the consent to participate was provided by 27 subjects (90%) in the “Social Point” group and by 21 subjects (70%) on the wait list group.

Main reason claimed for refusal to participate in both groups was poor timing.

### Inclusion criteria

Diagnosis: schizophrenic disorders (ICD-9295.xx) and other nonorganic psychoses (ICD-9298.xx). Gender and Age: both male and female subjects between 18 and 60 years of age.

Exclusion criteria were: Learning disabilities, organic mental disorders, drug induced-psychosis. Subject whose psychopathological conditions were not considered stable over the previous 3 months were also excluded from the sample.

### Consent to participate

Written informed consent was obtained before recruitment and a letter detailing the nature of the study was sent to the subjects’ General Practitioner. The study was approved by the local Ethics Committee of Modena.

### Social point activities and intervention

Social Point promotes meetings and assemblies aimed at planning social activities and events with the involvement of service users, family members, professionals and members of the larger community. In addition, Social Point supports and encourages users’ proposals and initiatives, as well as working towards transferring skills for the development of self-help groups.

Samples of activities promoted and supported by Social Point are: running and managing a radio station, carpentry and bike fixing workshops, magazine publishing, drama, poetry, painting, photography and cooking classes, community concerts.

Social Point intervention can be summarized in 5 defined phases aimed at personal recovery.

1. Assessment: patients referred by community mental health professionals are assessed together with the treating mental health team to evaluate suitability to participate with respect to current clients’ needs and motivation.
2. Orientation: service users are invited and supported to reflect about their interests, preferences (i.e. what he/she likes to do) and previous experiences. In the initial meetings, workers evaluate and consider the interest of patients to participate in the activities, which are offered according to resources available in the local community.
3. Coaching: service users are individually supported by a coach across the entire recovery path and helped to develop skills to maintain a healthy balance in life, rebuild meaningful relationships and overcome difficulties related to their mental illness and/or the everyday life experiences. The role of a coach is primarily to work in partnership with service users, who are considered the expert of his/her life and the one in charge to make decisions able to produce successful changes.
4. Implementation: Social Point workers activate the local community network and resources (e.g. cultural and recreational clubs, multi-media facilities, voluntary associations) that are not necessarily involved with or directed to mental health service users.
5. Reassessment: recovery-related outcomes are measured at the end of the program. It is important to point out that the end of the program does not necessarily means that the activities will be interrupted by the patients; on the contrary, their continuation without the supervision of “Social Point” remains the main goal of the project.

### Psychometric scales

All subjects were assessed by means of the following evaluation instruments:

- HoNOS/MHCT (Health of the Nation Outcome Scale/Mental Health Clustering Tool). Higher scores indicate higher level of disability, lower functioning and higher severity of psychopathology [37, 38]. For the purpose of the study, the first 12 items were combined into four dimensions: “behaviour” including (aggressiveness; non-accidental self-injury; problem drinking and drug taking), “impairment” (cognitive problems; physical illness or disability problems), “symptoms” (hallucinations and delusions; depressed mood; other mental and behavioural problems) and “social problems” (problems with relationships; problems with activities of daily living; problems with living conditions; problems with occupation and activities). For each dimension the scores were calculated adding the values of the single items, which may vary from 0 (absent) to 4 (severe).
- Global Assessment of Functioning (GAF) [39]. Scores are arranged in 10 levels of increasing severity, where the interval 91–100 indicates “superior” level of functioning and 1–10 points out the highest impairment.
- Measure of Activity in Psychosis [40]. The single item score is arranged in a gradient of severity ranging from 0 to 4 (the first indicating a severely impaired level of activity, whereas the latter corresponds to an overall adequate and independent engagement in activities of daily living). In this case, higher figures are indicative of a better level of activity.
- Rosenberg Self-Esteem Scale [41]. Which is a 10-item scale that measures global self-worth by measuring both positive and negative feelings about the self. All items are answered using a 4-point Likert scale format ranging from “strongly agree” to “strongly disagree”. Higher scores indicate higher self-esteem.
- Internalised Stigma of Mental Illness (ISMI) [42]. Scores were calculated in a four-categories method, according to Lysaker [27]: “Alienation”, “Stereotypes”, “Discrimination experiences” and “Social withdrawal”. The category “Resistance to stigma” was considered separately as it can be associated with less internalised stigma. Higher scores associate with higher levels of internalised stigma.
- World Health Organization quality of life assessment-BREF (brief version) (WHOQOL-BREF) [43]. For the present study four domain scores were derived: “Physical health”, “Psychological”, “Social relationships” and “Environmental”. The first two items (i.e. “Overall quality of life” and “Satisfaction with Health”) were listed separately as they are not counted in any of the previous domains. Higher scores denote a higher quality of life.

### Statistical analyses

A descriptive statistical analysis was first carried out to establish statistically significant differences between the two samples. For the comparison of frequencies, the Fisher’s exact test was used. Differences between the mean scores of the two groups were assessed by Wilcoxon-Mann-Whitney tests on the following areas: level of functioning (GAF), level of activity (Measure of Activity in Psychosis Scale), social functioning and severity of symptoms (HoNOS/MHCT), level of self-esteem (Rosenberg Self-Esteem Scale), quality of life (WHOQOL-BREF) and internalised stigma (ISMI). Moreover, possible differences in hospitalization rate and length of stay, during the period of the study, were investigated between the two groups and statistical significance was evaluated by means of the above tests.

Among subjects who completed “Social Point” program additional analyses were performed: to evaluate the association between variables of the program provided (i.e. duration of the program and number of activities per patient) and the scores at the evaluation instruments administered, Spearman’s rank correlation test was used. For all the analysis a difference of *p* < 0.05 was considered statistically significant.

Data collected were analyzed via SAS Enterprise Guide [2009].

## Results

Subjects who completed the “Social Point” program (SP) and those in the wait list (WL) did not show significant differences with respect to socio-demographic characteristics. The two groups were also homogeneous with respect to diagnoses (Table 1).

**Table 1:** Socio-demographic and clinical characteristics of subjects who completed Social Point program and subjects in the wait list

	Social Point *N* = 27	Wait list *N* = 21	*p*-value*
Gender = M	20 (74.1%)	14 (66.7%)	0.7502
Marital status			
Unmarried or divorced	23 (92.0%)	19 (90.5%)	0.9999
Married or in patnership	2 (7.4%)	2 (9.5%)	
Qualification			
Primary or no qualification	10 (37.0%)	9 (42.9%)	
Middle or high school	13 (48.2%)	12 (57.1%)	0.2087
University or higher degree	4 (14.8%)	0 (0.0%)	
Profession			
Occupied	8 (40.0%)	6 (35.3%)	
Unemployed	9 (45.0%)	5 (29.4%)	0.4037
Other non-professional condition	3 (15.0%)	6 (35.3%)	
Housing status			
Live alone	4 (16.7%)	1 (4.8%)	0.6772
Family-in-law	2 (8.3%)	3 (14.3%)	
Family of origin	16 (66.7%)	16 (76.2%)	
Residential accomodation	2 (8.3%)	1 (4.8%)	
Diagnosis			
Schizophrenic disorders	23 (85.2%)	19 (90.5%)	0.6830
Other nonorganic psychoses	4 (14.8%)	2 (9.5%)	

*Fisher’s exact test

The median duration of SP program was 548 days. During this period, the median number of social inclusion activities per patient was 4.

The analysis of GAF total score showed a higher level of functioning in those subjects who had completed SP program [SP mean = 67.4 (SD = 13.4) vs WL mean = 52.0 (SD = 17.5), *p* = 0.0006]; the SP group mean score fell within the “mild impairment” (70–61) range, whereas subjects in the WL group presented a “moderate impairment” (60–51). SP subjects reported higher scores at the Measure of Activity in Psychosis [SP = mean 2.8 (SD = 0.9) vs WL mean = 2.2 (SD = 0.9), *p* = 0.0129]. The HoNOS/MHCT showed a significant difference between the two groups, with regards to the “Social problems” subscale, which presented more favourable scores in SP subjects. On the other hand, the level of self-esteem observed, as measured by means of the Rosenberg scale, resulted higher in the WL group compared with SP group [SP mean = 25.6 (SD = 1.8) vs WL mean = 26.8 (SD = 2.4), *p* = 0.0231] (Table 2).

**Table 2:** Outcome measures of subjects who completed Social Point program and subjects in the wait list

Psychometric scales	Program	N	Average score	C.I. 95%	St. D.	*p*-value*
GAF	Social Point	27	67.4	62.1–72.7	13.4	0.0006
	Wait list	21	52.0	44.7–58.9	17.5	
Level of activity	Social Point	27	2.8	2.5–3.2	0.9	0.0129
	Wait list	21	2.2	1.8–2.6	0.9	
Honos Behaviour	Social Point	27	0.7	0.3–1.0	0.9	0.3118
	Wait list	21	0.6	0.2–1.0	0.9	
Honos Impairment	Social Point	27	1.9	1.2–2.3	1.9	0.1705
	Wait list	21	1.8	0.8–2.1	1.8	
Honos Symptoms	Social Point	27	3.9	2.2–5.7	4.6	0.3002
	Wait list	21	4.4	2.6–4.8	5.6	
Honos Social problems	Social Point	27	4.6	3.2–6.1	3.3	0.0352
	Wait list	21	6.2	4.8–7.4	3.4	
Rosenberg	Social Point	27	25.6	25.0–26.3	1.8	0.0231
	Wait list	21	26.8	25.8–28.1	2.4	

*Wilcoxon-Mann-Whitney non-parametric test

Internalised stigma (ISMI) and quality of life (WHOQOL-BREF) scores were more favourable among the WL group compared with SP subjects. Significant differences were found within the following domains: “Discrimination experiences” and “Social withdrawal” at the ISMI scale (Table 3) and “Social relationships” and “Psychological” at the WHOQOL-BREF (Table 4).

**Table 3:** ISMI scores of patients who completed Social Point program and subjects in the wait list

ISMI scale		N	Average score	C.I. 95%	St. D.	*p*-value*
Total	Social Point	27	2.2	2.1–2.4	0.4	0.0212
	Wait list	21	2.0	2.0–2.2	0.3	
Domains						
Alienation	Social Point	27	2.2	2.0–2.4	0.6	0.0989
	Wait list	21	2.0	1.8–2.2	0.4	
Stereotype	Social Point	27	2.1	1.9–2.2	0.4	0.0796
	Wait list	21	1.9	1.8–2.1	0.3	
Discrimination experience	Social Point	27	2.3	2.1–2.5	0.5	0.0006
	Wait list	21	1.9	1.8–2.1	0.3	
Social withdrawal	Social Point	27	2.3	2.1–2.5	0.5	0.0179
	Wait list	21	2.0	1.8–2.2	0.5	
Stigma Resistance	Social Point	27	2.7	2.5–2.9	0.5	0.1597
	Wait list	21	2.6	2.4–2.8	0.4	

*Wilcoxon-Mann-Whitney non-parametric test

**Table 4:** WHOQOL-BREF scores of patients who completed Social Point program and subjects in the wait list

WHOQOL scale		N	Average score	C.I. 95%	St. D.	*p*-value*
Overall quality of life	Social Point	27	3.4	3.0–3.8	1.0	0.4001
	Wait list	21	3.5	3.0–3.9	1.0	
Satisfaction with Health	Social Point	27	3.4	3.0–3.9	1.1	0.1769
	Wait list	21	3.8	3.3–4.2	0.9	
Domains						
Physical health	Social Point	27	24.0	21.8–26.2	5.5	0.1152
	Wait list	21	26.1	24.0–28.2	4.6	
Psychological	Social Point	27	18.7	17.1–20.3	4.0	0.0424
	Wait list	21	20.6	18.9–22.3	3.7	
Social relationships	Social Point	27	8.7	7.8–9.6	2.3	0.0027
	Wait list	21	10.9	9.8–11.9	2.2	
Environment	Social Point	27	25.3	23.0–27.6	5.8	0.0639
	Wait list	21	27.9	25.9–29.8	4.6	

*Wilcoxon-Mann-Whitney non-parametric test

No significant difference was found between the two groups with respect to admission rate and length of stay. The admission rate was 19% in the WL group and 7.4% in the SP group (*p* = 0.3827). The median length of stay was 48.5 days in the SP group and 27.5 days in the WL group (*p* = 0.2994).

**Fig. 1:**
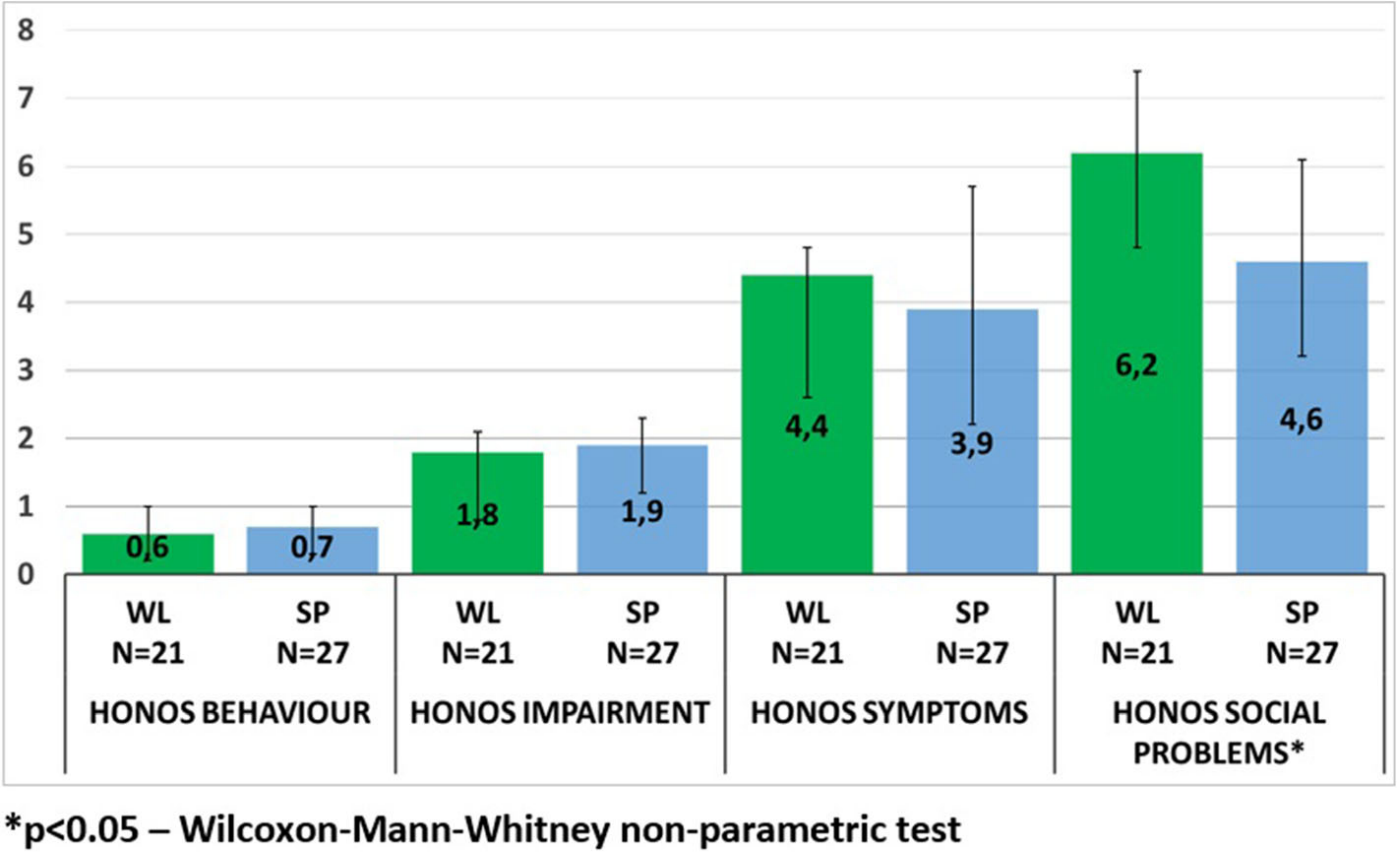
HoNOS/MHCT subscales mean scores

Correlations analyses between variables of “Social Point” program and the assessment measures showed a significant dose-effect relationship between the number of social inclusion activities per patient and the score on “Social problems” (Spearman’s rank correlation = − 0.5368, *p* = 0.0039) and “Symptoms” (Spearman’s rank correlation = − 0.3926, *p* = 0.00428) MHCT/MHCT (Fig. 1). Higher number of social inclusion activities was associated with an improvement in clinical and social dimensions.

The number of social inclusion activities per patient was also associated with better outcomes at the GAF (Spearman’s rank correlation = 0.4026, *p* = 0.0373) and the Measure of Activity in Psychosis scale scores (Spearman’s rank correlation = 0.5159, *p* = 0.0059) (Table 5).

**Table 5:** Spearman’s rank correlations between outcome measures and variables of Social Point program

	Duration of program	Number of social inclusion activities
GAF	0.3629	0.4026*
Level of activity	0.3744	0.5159**
Honos Behaviour	0.2116	−0.0010
Honos Impairment	−0.2877	−0.3542
Honos Symptoms	−0.3777	−0.3926*
Honos Social problems	−0.2065	−0.5368**

*p < 0.05

**p < 0.01

With regard to the number of social inclusion activities per patient, a significant correlation was found on the HoNOS/MHCT items: “Cognitive problems”, “Problems with activities of daily living”, “Problems with living conditions”, “Strong and unreasonable beliefs in non-psychotic disorders only” and the domain “Stereotype” at the ISMI scale.

No correlation was found between the scores on the items of the ISMI scale, WHOQOL-BREF, the Rosenberg Self-Esteem Scale and number of social inclusion activities per patient.

Finally, a correlation was found between a longer duration of the program and the item 6 (Hallucinations and delusions) at the HoNOS/MHCT (Spearman’s rank correlation = − 0.5368, *p* = 0.0039).

## Discussion

Overall, the results of the study support the evidence that the implementation of social inclusion programs in people suffering from severe mental illness determine significant improvement across several psychosocial dimensions [13, 17, 18]. Indeed, the comparison between the two groups showed that subjects who completed “Social Point” program presented lesser impairment in different areas of social functioning (GAF and HoNOS/MHCT) and had better scores in terms of level of activity (Measure of Activity in Psychosis).

However, in spite of the objective and substantial advantages found in the areas of functioning and activity, the results of self-administered scales investigating self-esteem (Rosenberg), perceived quality of life (WHOQOL-BREF) and internalised stigma (ISMI) indicated a worse profile in the SP group compared with WL subjects. In this respect, it can be hypothesized that subjects participating to social inclusion programs are implicitly pushed to step out of their “comfort zone” and exposed to certain environmental stressors which would not normally encounter, if left marginalized from the community. Moreover, they may end up internalizing their social isolation and accept it as a “natural” condition, whereas the efforts requested by rehabilitation programs may be frustrating at times. This process may represent an explanation for the apparently contradictory nature of the results observed at the self-administered scales. However, longitudinal analyses are necessary to clarify whether the self-perception of quality of life and internalised stigma, which in our samples were significantly different on several dimensions (i.e.: “Social relationships” and “Psychological” at WHOQOL-BREF and “Discrimination experiences” and “Social withdrawal” at ISMI), may gradually decrease overtime due to the social skills and opportunities gained via social inclusion interventions.

Results also stress the importance of intensive and enduring social inclusion interventions. The analysis carried out among subjects who completed “Social Point” program, showed that those who were involved in a higher number of social inclusion activities had better scores on clinical dimensions and social functioning. This result reinforces the notion that interventions aimed at individual recovery cannot be, by any means, a standard procedure but should necessarily be individualised. The interventions provided by “Social Point” follow this principle and involve different agencies in the community, which increases the possibility of providing patients with a wide array of activity choices and multiple individualized projects.

Indeed, higher number of activities per patient associated with better outcomes in terms of cognitive functioning and better clinical presentation such as the HoNOS/MHCT item “Strong and Unreasonable beliefs”, which is consistent with the growing evidence that treatment of cognition represents a pivotal step in severe mental illness rehabilitation [44]. A higher number of activities was also found to be associated with better scores at the HoNOS/MHCT item “Activities of daily living” suggesting potential benefits in terms of problems with basic tasks such as eating, washing, dressing, but also requiring more complex skills such as budgeting, organising where to live, occupational and mobility planning and self-development.

Finally, it can be postulated that adequate duration of social inclusion activities may positively influence several outcomes, not necessarily confined to those within the social domain. As a matter of fact, subjects who stayed on “Social Point” program for longer, showed better HoNOS/MHCT scores with respect to psychopathology (i.e. item 6 “Hallucinations and delusions”).

The study has some limitations that need to be acknowledged. It was conducted in one specific geographic area of Northern Italy and it is uncertain whether the results would translate to similar population in other countries. The cross-sectional design of the study does not allow to draw any definitive conclusion about the cause-effect relation of the variables considered. Despite the high similarity of the two groups, we cannot exclude that subjects who were given priority to the “Social Point” program, over those on the wait list, were already more amenable and willing to participate due to a less severe impairment of social functioning and/or milder clinical picture. Further investigation with larger sample and longitudinal design is warranted to replicate the result of this study and clarify the role of psychosocial intervention in severe mental illness.

## Conclusion

This study advocates structured social inclusion programmes for people suffering from psychosis, suggesting that they may be an effective intervention. Moreover, in consideration of the observed importance of providing personalised interventions with a wide array of activities and lengthy programmes, it would be highly desirable that the availability of such treatment option became a routine practice for community mental health services.

## Abbreviations

GAF: Global Assessment of Functioning
HoNOS/MHCT: Health of the Nation Outcome Scale/Mental Health Clustering Tool
ISMI: Internalised Stigma of Mental Illness
SAS: Statistical Analysis Software
SP: Social Point
WHOQOL-BREF: World Health Organization quality of life assessment-BREF (brief version)
WL: Waiting List

## References

[1] Huxley P, Evans S, Madge S, Webber M, Burchardt T, McDaid D, Knapp M (2012). Development of a social inclusion index to capture subjective and objective life domains (phase II): psychometric development study. Health Technol Assess.

[2] Gaite L, Vazquez-Barquero JL, Borra C, Ballesteros J, Schene A, Welcher B (2002). Quality of life in patients with schizophrenia in five European countries: the EPSILON study. Acta Psychiatr Scand.

[3] Brundtland GH (2001). From the World Health Organization. Mental health: new understanding, new hope. JAMA.

[4] Harvey CA, Jeffreys SE, McNaught AS, Blizard RA, King MB (2007). The Camden schizophrenia surveys. III: five-year outcome of a sample of individuals from a prevalence survey and the importance of social relationships. Int J Soc Psychiatry.

[5] Thornicroft G, Strathdee G, Phelan M, Holloway F, Wykes T, Dunn G (1998). Rationale and design. PRiSM psychosis study I. Br J Psychiatry.

[6] Walsh E, Leese M, Taylor P, Johnston I, Burns T, Creed F (2002). Psychosis in high-security and general psychiatric services: report from the UK700 and special hospitals’ treatment resistant schizophrenia groups. Br J Psychiatry.

[7] Stilo SA, Di Forti M, Mondelli V, Falcone AM, Russo M, O'Connor J (2013). Social disadvantage: cause or consequence of impending psychosis?. Schizophr Bull.

[8] Valtorta NK, Kanaan M, Gilbody S, Ronzi S, Hanratty B (2016). Loneliness and social isolation as risk factors for coronary heart disease and stroke: systematic review and meta-analysis of longitudinal observational studies. Heart.

[9] Holt-Lunstad J, Smith TB, Baker M, Harris T, Stephenson D (2015). Loneliness and social isolation as risk factors for mortality: a meta-analytic review. Perspect Psychol Sci.

[10] Anderson K, Laxhman N, Priebe S (2015). Can mental health interventions change social networks? A systematic review. BMC Psychiatry.

[11] Killaspy H, White S, Lalvani N, Berg R, Thachil A, Kallumpuram S (2014). The impact of psychosis on social inclusion and associated factors. Int J Soc Psychiatry.

[12] Becker T, Thornicroft G, Leese M, McCrone P, Johnson S, Albert M (1997). Social networks and service use among representative cases of psychosis in South London. Br J Psychiatry.

[13] Brennaman L, Lobo ML (2011). Recovery from serious mental illness: a concept analysis. Issues Ment Health Nurs.

[14] Cohen S (2004). Social relationships and health. Am Psychol.

[15] Uchino BN (2006). Social support and health: a review of physiological processes potentially underlying links to disease outcomes. J Behav Med.

[16] Buchanan J (1995). Social support and schizophrenia: a review of the literature. Arch Psychiatr Nurs.

[17] Webber M, Fendt-Newlin M (2017). A review of social participation interventions for people with mental health problems. Soc Psychiatry Psychiatr Epidemiol.

[18] Choenarom C, Williams RA, Hagerty BM (2005). The role of sense of belonging and social support on stress and depression in individuals with depression. Arch Psychiatr Nurs.

[19] Pinto RM (2006). Using social network interventions to improve mentally ill Clients’ well-being. Clin Soc Work J.

[20] Layard R, Clark D, Knapp M, Mayraz G. Cost-benefit analysis of psychological therapy. London: Centre for Economic Performance London School of Economics and Political Science. 2007. ISBN 978 0 85328 094 1.

[21] Huggett C, Birtel MD, Awenat YF, Fleming P, Wilkes S, Williams S, Haddock G. A qualitative study: experiences of stigma by people with mental health problems. Psychol Psychother. 2018. 10.1111/papt.12167. 10.1111/papt.12167 29345416

[22] Berry C, Greenwood K. Direct and indirect associations between dysfunctional attitudes, self-stigma, hopefulness and social inclusion in young people experiencing psychosis. Schizophr Res. 2017. 10.1016/j.schres.2017.06.037. [Epub ahead of print]. 10.1016/j.schres.2017.06.037 28693753

[23] Horan WP, Rassovsky Y, Kern RS, Lee J, Wynn JK, Green MF. Further support for the role of dysfunctional attitudes in models of real-world functioning in schizophrenia. J Psychiatr Res. 2010;44(8):499–05. 10.1016/j.jpsychires.2009.11.001. 10.1016/j.jpsychires.2009.11.001 PMC3414437 20006849

[24] Yanos PT, Lysaker PH, Roe D (2010). Internalised stigma as a barrier to improvement in vocational functioning among people with schizophrenia-spectrum disorders. Psychiatry Res.

[25] Corrigan PW, Larson JE, Rusch N (2009). Self-stigma and the “why try” effect: impact on life goals and evidence-based practices. World Psychiatry.

[26] Yanos PT, Roe D, Markus K, Lysaker PH (2008). Pathways between internalised stigma and outcomes related to recovery in schizophrenia spectrum disorders. Psychiatr Serv.

[27] Lysaker PH, Roe D, Yanos PT (2007). Toward understanding the insight paradox: internalised stigma moderates the association between insight and social functioning, hope, and self-esteem among people with schizophrenia spectrum disorders. Schizophr Bull.

[28] Lysaker PH, Davis LW, Warman DM, Strasburger A, Beattie N (2007). Stigma, social function and symptoms in schizophrenia and schizoaffective disorder: associations across 6 months. Psychiatry Res.

[29] Cechnicki A, Angermeyer MC, Bielanska A (2011). Anticipated and experienced stigma among people with schizophrenia: its nature and correlates. Soc Psychiatry Psychiatr Epidemiol.

[30] Lysaker PH, Tunze C, Yanos PT, Roe D, Ringer J, Rand K (2012). Relationships between stereotyped beliefs about mental illness, discrimination experiences, and distressed mood over 1 year among persons with schizophrenia enrolled in rehabilitation. Soc Psychiatry Psychiatr Epidemiol.

[31] Ucok A, Brohan E, Rose D, Sartorius N, Leese M, Yoon CK (2012). Anticipated discrimination among people with schizophrenia. Acta Psychiatr Scand.

[32] Rusch N, Corrigan PW, Powell K, Rajah A, Olschewski M, Wilkniss S (2009). A stress-coping model of mental illness stigma: II. Emotional stress responses, coping behavior and outcome. Schizophr Res.

[33] Sibitz I, Unger A, Woppmann A, Zidek T, Amering M (2011). Stigma resistance in patients with schizophrenia. Schizophr Bull.

[34] Mossabir R, Morris R, Kennedy A, Blickem C, Rogers A (2015). A scoping review to understand the effectiveness of linking schemes from healthcare providers to community resources to improve the health and well-being of people with long-term conditions. Health Soc Care Community.

[35] Fung KM, Tsang HW, Cheung WM (2011). Randomized controlled trial of the self-stigma reduction program among individuals with schizophrenia. Psychiatry Res.

[36] MacInnes DL, Lewis M (2008). The evaluation of a short group programme to reduce self-stigma in people with serious and enduring mental health problems. J Psychiatr Ment Health Nurs.

[37] Wing JK, Beevor AS, Curtis RH, Park SB, Hadden S, Burns A (1998). Health of the Nation Outcome Scales (HoNOS/MHCT). Research and development. Br J Psychiatry.

[38] Department of Health (2013). The mental health clustering booklet v 3.0 2013/14.

[39] American Psychiatric Association - APA. Diagnostic and statistical manual of mental disorders, 4th Edition. Washington: The American Psychiatric Association. 1994

[40] Jolley S, Garety P, Dunn G, White J, Aitken M, Challacombe F (2005). A pilot validation study of a new measure of activity in psychosis. Soc Psychiatry Psychiatr Epidemiol.

[41] Rosenberg M (1965). Society and the adolescent self-image.

[42] Ritsher JB, Otilingam PG, Grajales M (2003). Internalised stigma of mental illness: psychometric properties of a new measure. Psychiatry Res.

[43] The WHOQOL Group (1998). Development of the World Health Organization WHOQOL-BREF quality of life assessment. Psychol Med.

[44] Spaulding WD, Sullivan ME (2016). Treatment of cognition in the schizophrenia Spectrum: the context of psychiatric rehabilitation. Schizophr Bull.

